# Expression of Concern: *Mansonella*, including a Potential New Species, as Common Parasites in Children in Gabon

**DOI:** 10.1371/journal.pntd.0010921

**Published:** 2022-12-13

**Authors:** The PLOS Neglected Tropical Diseases Editors

This article [1] has been identified as one of a series of submissions for which we have concerns about the reported research ethics approval information and the article’s adherence to PLOS research ethics policies.

PLOS will be investigating these concerns in accordance with COPE guidance and journal policies. Meanwhile, the *PLOS Neglected Tropical Diseases* Editors issue this Expression of Concern.
